# The combined effect of determinants on coverage of intermittent preventive treatment of malaria during pregnancy in the Kilombero Valley, Tanzania

**DOI:** 10.1186/1475-2875-11-S1-O17

**Published:** 2012-10-15

**Authors:** Karin Gross, Sandra Alba, Joanna Schellenberg, Flora Kessy, Iddy Mayumana, Brigit Obrist

**Affiliations:** 1 Swiss Tropical and Public Health Institute, Basel, Switzerland; 2 University of Basel, Basel, Switzerland; 3 lfakara Health Institute, Dar es Salaam, Tanzania; 4 London School of Hygiene and Tropical Medicine, London, UK; 5 University of Basel, Institute of Anthropology, Basel, Switzerland

## Background

Intermittent preventive treatment during pregnancy (IPTp) at routine antenatal care (ANC) clinics is an important and efficacious intervention to reduce adverse health outcomes of malaria infections during pregnancy. However, coverage for the recommended two IPTp doses is still far below the 80% target in Tanzania. This paper investigates the combined impact of pregnant women’s timing of ANC attendance, health workers’ IPTp delivery and different delivery schedules of national IPTp guidelines on IPTp coverage.

## Materials and methods

Data on pregnant women’s ANC attendance and health workers’ IPTp delivery were collected from ANC card records during structured exit interviews with ANC attendees and through semi-structured interviews with health workers in south-eastern Tanzania. Women’s timing of ANC visits and health worker’s timing of IPTp delivery were analyzed in relation to the different national IPTp schedules and the outcome on IPTp coverage was modelled.

## Results

Among all women eligible for IPTp, 79% received a first dose of IPTp and 27% were given a second dose. Although pregnant women initiated ANC attendance late, their timing was in line with the national guidelines recommending IPTp delivery between 20-24 weeks and 28-32 weeks of gestation. Only 15% of the women delayed to the extent of being too late to be eligible for a first dose of IPTp. Less than 1% of women started ANC attendance after 32 weeks of gestation. During the second IPTp delivery period health workers delivered IPTp to significantly less women than during the first one (55% vs. 73%) contributing to low second dose coverage. Simplified IPTp guidelines for front-line health workers as recommended by WHO could lead to a 20 percentage point increase in IPTp coverage.

## Conclusions

This study suggests that facility and policy factors are greater barriers to IPTp coverage than women’s timing of ANC attendance. To maximize the benefit of the IPTp intervention, revision of existing guidelines is needed. Training on simplified IPTp messages should be consolidated as part of the extended antenatal care training to change health workers’ delivery practices and increase IPTp coverage. Pregnant women’s knowledge about IPTp and the risks of malaria during pregnancy should be enhanced as well as their ability and power to demand IPTp and other ANC services.

